# Subsequent Emergency Department Visits in Geriatric Mild Traumatic Brain Injury: Relationship with Fall, Payor, and Discharge Outcome

**DOI:** 10.3390/healthcare13111236

**Published:** 2025-05-23

**Authors:** Carrie A. Barrett, Mark G. Goetting, Rob Lyerla, Kieran Fogarty

**Affiliations:** 1 Doctor of Physical Therapy Program, Western Michigan University, Kalamazoo, MI 49008, USA; 2 Homer Stryker M. School of Medicine, Western Michigan University, Kalamazoo, MI 49008, USA; goetting@live.com; 3 Interdisciplinary Health Sciences, Western Michigan University, Kalamazoo, MI 49008, USA; rob.lyerla@wmich.edu (R.L.); kieran.fogarty@wmich.edu (K.F.)

**Keywords:** traumatic brain injury, concussion, emergency department, geriatric, falls, discharge, payor, insurance, health services

## Abstract

**Background/Objectives**: Older adults (ages ≥ 65) have experienced longer recovery, decreased independence in self-care, and reduced quality of life after diagnosis of mild traumatic brain injury (mTBI). Although the sequela following mTBI has also generated higher healthcare costs in older adults, the research on associations returning to the emergency department (ED) has been limited. This study explored subsequent mild traumatic brain injury (mTBI-S) ED visit relationships among older adult populations, fall injuries, payors, and discharge outcomes. **Methods**: The design was a population-based cross-sectional study using data from the 2018 Nationwide Emergency Department Sample (NEDS). The study sample size was 4932. Descriptive analysis and correlation analysis described characteristics of people with subsequent mTBI visits. Logistic regressions and odds ratios ascertained the relationship between subsequent mTBI visits and the predictor variables of age, fall injury, payors (Medicare, Medicaid, Private, and other), and the outcome variable of healthcare services. **Results**: Falls and referrals to healthcare service associations were significant (*p* < 0.001, X^2^_1_ = 123.6). The association between Medicare and referral to healthcare service visits was also significant (*p* < 0.001, X^2^_3_ = 1059.9). The odds ratio in populations aged ≥65 (OR 4.172, *p* < 0.001, CI 95% 3.427, 5.079), falls (OR 3.847, *p* < 0.001, CI 95% 2.649, 5.587), and Medicare (OR 4.492, *p* < 0.001, CI 95% 1.273, 2.106) had an increased probability of referral to healthcare services. **Conclusions**: Geriatric populations, falls, and Medicare carriers had an increased probability of healthcare service referral upon readmission to the ED for persistent symptoms after mTBI. Research on geriatric populations and post-mTBI medical monitoring may inform ED discharge models.

## 1. Introduction

Individuals diagnosed with traumatic brain injuries made up 4.3 million U.S. emergency department (ED) admissions in 2018 [1]. Among these, mild traumatic brain injuries (mTBI) constituted 90% of all traumatic brain injury cases according to Maas et al., 2022 [2]. An mTBI, or concussion, has been defined as a mechanical hit to the head, rapid head movement relative to the body, or blast injury [3]. Although mTBI awareness and prevention have been developing in the research, increased numbers of admissions have been reported. In a recent trend analysis, Shah et al. reported a three-fold increase from 1997 to 2019 in mTBI U.S. ED visits, from 82,103 (1997) to 261,722 (2019) (R^2^ = 0.9036, *p* < 0.001) [4].

The “mild” classification of mTBI has masked the significant impact of this condition in older adult populations [5,6,7,8,9,10]. In the most current research on mTBI and healthcare costs, older adults had an increased occurrence of mTBI, which increased the cost burden on the U.S. healthcare system ($22 million per visit for individuals aged ≥65 compared to $11 million in those younger than age 65) [5]. Additionally, investigators reported older adults experienced a longer recovery (44% of participants after 6 months) [6,7], decreased performance of activities of daily living or self-care [9,10], and poorer quality of life secondary to mTBI when compared to populations younger than 65 [10].

According to Varriano et al., fall-related mechanisms of injury were more common in individuals aged at or over 65 compared to other age groups among Canadian participants with lasting symptoms after mTBI (OR 11.11, CI 5.56–25.0) [11]. Similarly, Shah et al. reported mechanisms of injury on a 1997–2019 yearly concussion estimate and stated the most common mechanism, fall to the floor, was observed in those aged ≥65. However, details of whether this population returned to access the ED in subsequent visits were not described [4]. There has been limited research in U.S. mTBI populations aged ≥65 and associations with fall mechanism of injury for those returning to the ED for subsequent visit admissions.

Researchers have reported that payor source was influential in ED discharge considerations and access to follow-up care [12,13]. The research, however, was limited regarding the associations between payor source and subsequent visits for individuals aged ≥65 with mTBI [14]. Seabury et al. reported that the insurance source had not been associated with healthcare service utilization after discharge with mTBI; however, the study was not specific to those aged ≥65 [14]. According to the Centers for Disease Control and Prevention National Health Statistics Report published in 2021, health insurance coverage among those aged ≥65 showed that Medicare covered only 13.6% of patients, Medicare Advantage 20.0%, Private insurance 40.9%, with the remainder being covered by “other” and dual-eligible plans [15]. This was an unusual finding, as Medicare being the primary payor for those aged ≥65 was a common assumption [16]. Given the diversity in coverage and the lack of age-specific data, further investigation was needed to understand if payor source was associated with discharge planning among older adults (aged ≥65) and in subsequent mTBI visits.

Additionally, there were contrasting study results in those aged ≥65 with mTBI and ED discharge locations [17,18,19]. Vadlamani et al., in a Maryland retrospective study, reported that 52% of those aged ≥65 with mTBI were discharged to an inpatient facility and 48% were discharged home without services [17]. Conversely, Thomson et al. reported that older age was linked to greater use of skilled nursing facilities and noted a lack of coordinated services for geriatric patients with mTBI after ED discharge [18]. In yet another study by Borgen et al., 93% of all mTBI ED visits were discharged home without healthcare services [19]. Seabury et al. and Caplan et al. noted a lack of large-sample research on populations aged ≥65 with mTBI, specifically regarding the associations between payor source and discharge location. They emphasized the need for future studies due to the aging U.S. population [14,20]. Therefore, this study aimed to explore subsequent mTBI ED visits’ (mTBI-S) relationships with variables of geriatric populations (ages ≥ 65), fall-related injuries, payor sources, and associations with ED discharge outcomes. By analyzing these variables together, the study intended to provide insights into older adult populations, fall-reported injuries, payor source consistency, and discharge referral patterns in those returning to the ED after mTBI. Understanding these patterns may ultimately reduce unnecessary visits, guide policy to improve continuity of care, and lower healthcare costs in older adults.

## 2. Materials and Methods

### 2.1. Study Design

The present study was a secondary data analysis that used a cross-sectional design. The study was approved by the Western Michigan University Human Subjects Internal Review Board (#2023-175), and the National Emergency Department Sample (NEDS) Data User Agreement was signed.

### 2.2. Participants/Data Source

The NEDS, overseen by the Healthcare Cost and Utilization Project (HCUP) through the Agency for Quality Research in Healthcare (AHQR), has been the largest U.S. public access ED dataset. The NEDS dataset has been used to determine population trends, costs, and medical service usage, and to guide healthcare policy changes [21]. The present research study used the most current dataset available (2018) at the time of the study. The NEDS 2018 dataset represented all regions throughout the U.S., including 990 hospitals across 37 states [21]. In the HCUP NEDS 2018 explanation of data collection, based on the data collected by the U.S. Census Bureau, 82.8% of the total U.S. resident population and 82.2% of all U.S. ED visits were included in the dataset [21].

Each ED visit had associated diagnosis codes, patient characteristics, and mechanisms of injury. The NEDS identified the diagnosis code for mTBI-S as INJ045 (concussion without loss of consciousness, subsequent encounter) using the clinical classification software refined (CCSR) code [21]. The INJ045 code corresponded to the 10th revision of the International Statistical Classification of Diseases and Related Health Problems (ICD-10) code S060X0D [21]. The predictor variables included age, self-reported sex, fall injury, and payor source. The variable of age, originally treated as a continuous variable, was dichotomized into two groups: older adults (ages ≥ 65) and those younger than 65. While dichotomizing continuous variables may reduce statistical power and obscure validity within age groups, this approach was employed to specifically examine model performance within an older adult population, an age group of particular clinical relevance in mTBI research [22,23]. This decision allowed for targeted subgroup analysis, facilitating a clearer understanding of model outcomes relevant to age-related vulnerabilities and healthcare planning for geriatric populations. Fall-related injury was a code assigned at ED admission as a mechanism of injury and was dichotomized as “yes—fall-related injury” versus “no—all other mechanisms of injury”. The payor variable derived from the dataset was listed as the primary insurer for the ED service visit and included Medicare, Medicaid, Private, and other. The Medicare and Medicaid variables each included fee-for-service and managed care plans. The Private insurance variable included Blue Cross, commercial carriers, private health maintenance organizations, and preferred provider organizations. The category “other” included workers’ compensation, the Civilian Health and Medical Program of the Uniformed Services (CHAMPUS), the Civilian Health and Medical Program of the Department of Veterans Affairs (CHAMPVA), Title V, and other government programs [21]. Healthcare service referral indicated the disposition of the patient at discharge from the emergency department and included the following categories: discharge to home/“routine” (included against medical advice), transfer to a short-term hospital stay, transfer to other health services (skilled nursing facility, intermittent care-which included inpatient rehabilitation), home healthcare, admission to this hospital, died in the ED, and missing [21].

### 2.3. Statistical Methods

#### 2.3.1. Inclusion and Exclusion Criteria

The inclusion criteria exclusively consisted of diagnosis codes for mTBI-S and included the coded variables of age, sex, fall mechanism of injury, payor source, and discharge outcome. The exclusion criteria consisted of data showing ≤10 visits in the dataset (due to potential breach of confidentiality per the NEDS terms of Data Use Agreement guidelines), diagnosis codes in the original dataset file that were not mTBI-S, and details about mechanisms of injury other than falls (codes delineating blast injury, assault, etc.).

#### 2.3.2. Statistical Analysis

The study sample size for the full dataset was n = 4932. The statistical analysis included descriptive statistics to compute the visit count and visit characteristics. Chi-squared tests with degrees of freedom (X^2^_df_) assessed associations between categorical variables (age, sex, fall injury, payor source) and group membership (healthcare service, home). The confidence interval was established as 95% (*p* < 0.05).

The research question was whether older adults (≥65 vs. <65), self-reported sex (female vs. male), fall-related injury (yes vs. no), and payor sources (Medicare, Medicaid, Private, or other) were associated with an increased probability of referral to healthcare services (health services vs. home) for people with subsequent mTBI ED admissions. A logistic regression analysis was performed to evaluate the relationship between selected predictor variables and the discharge outcome of referral to healthcare services. The predictor variables included age (≥65, <65), sex (female, male), fall injury (yes, no), and payor source (Medicare, Medicaid, Private, or other). The outcome variable was defined as referral to healthcare services (including short-term hospital, skilled nursing home, inpatient rehabilitation, admission to this hospital, or home healthcare) versus no referral (routine discharge to home or discharge against medical advice). The odds ratio (OR) and model fit statistics were used to interpret the results. Collinearity diagnostics were conducted to ensure the independence of the predictor variables. These diagnostics included correlations, collinearity of tolerance, variance inflation factor (VIF), and variance proportions. The collinearity diagnostics checked for dependence among the predictor variables and ensured their isolated associations with the outcome variable.

#### 2.3.3. Subgroup Analysis: Older Adults

The dataset of mTBI-S was further stratified to those aged ≥65 (n = 1793) to assess whether the associations observed in the full sample were consistent within the older adult subgroup. Descriptive statistics were used to highlight visit counts and percentages of the sample set. The refined research question examined whether self-reported sex (female vs. male), fall-related injury (yes vs. no), and payor sources (Medicare, Medicaid, Private, or other) were associated with an increased probability of referral to healthcare services (healthcare services vs. home) among older adult populations (aged ≥65) with subsequent mTBI ED admissions. The logistic regression stratified to those aged ≥65 was conducted using the predictor variables of sex (female or male), fall injury (yes or no), and payor source (Medicare, Medicaid, Private, or other), and the outcome variable of healthcare service referral (health services or home). The odds ratio (OR) and model fit were reviewed for this model.

## 3. Results

Descriptive statistics were used to highlight the study characteristics of the NEDS database (Table 1).

The visit counts coded for falls as a mechanism of injury were extremely fewer (4.2%) than all other injuries (95.8%). The payor-coded categories displayed the highest percentage of billed services to Medicare (37.4%) and the lowest percentage of billed services to the “other” category of self-pay, no charge, other insurance (14%), with only five visits coded as missing (0.1%). The discharge outcomes were slightly higher in visits coded as being referred to home/no services (59.1%) than those coded as referral to healthcare services (40.9%), with only three coded as missing (0.1%) in referrals upon discharge. Collinearity studies were calculated, and the variables of age, sex, falls, and payor source were found not to meet the criteria for collinearity (Appendix A).

Chi-squared analyses were performed for variables of age (≥65, <65), sex (female or male), falls (yes or no—other than falls), and payors (Medicare, Medicaid, Private, and other) by discharge outcomes (home/no services or healthcare services) (Table 2).

The variables of age ≥ 65 (*p* < 0.001, X^2^_1_ = 1142.2) and female sex (*p* < 0.001, X^2^_1_ = 46.8) were associated with healthcare services (Table 2). The results of the variable for fall injury with healthcare services were also associated with a large X^2^ (*p* < 0.001, X^2^_1_ = 123.6) (Table 2). The payor source findings indicated that Medicare was associated with healthcare services and resulted in a large X^2^ (*p* < 0.001, X^2^_3_ = 1059.9) (Table 2). Conversely, Medicaid, Private, and other payor sources were not associated with referrals to healthcare services.

The results of the logistic regression analysis presented in Table 3 suggested that subjects who were aged ≥65, of female sex, who reported a fall injury, and who reported having Medicare, Medicaid, and Private insurance payor sources were associated with discharge to healthcare services (*p* < 0.001). The OR associated with age ≥ 65 was 4.172 (CI 95% 3.427, 5.079), indicating a 317% increase in the probability of referral to healthcare services. The OR associated with a reported fall injury was 3.847 (CI 95% 2.649, 5.587), indicating a 285% increase in the probability of referral to healthcare services. The OR associated with the reported payor (Medicare) was 4.492 (CI 95% 3.423, 5.895), indicating a 349% increase in the probability of being referred to healthcare services.

The model fit for this regression model of discharge outcomes for subjects aged ≥65, of female sex, with fall injuries and different payor variables was significant (*p* < 0.001), and the Nagelkerke R Square was 0.34, indicating 34% variability in the outcome data that can be explained (Table 4).

Further analysis was stratified to a subgroup of subjects aged ≥65 to determine consistency within subsequent mTBI ED admissions. Compared to the overall dataset of m-TBI-S, the stratified sample’s (n = 1793) characteristics contained similar percentages for female (44.2%) and male (55.8%) subjects (Table 5). The report of fall injury was slightly higher (7.5%) in this subgroup of people aged ≥65. The payor source percentages in the stratified sample differed, resulting in a higher percentage of Medicare as the primary payor source (87.3%). Collinearity studies were calculated, and the variables of sex, falls, and payor source were found not to meet the criteria for collinearity (Appendix B).

The results of the logistic regression analysis for the stratified sample had consistent findings compared to the overall mTBI-S sample. Subjects aged ≥65, of female sex (*p* < 0.001), with fall injury (*p* < 0.001) and Medicare (*p* = 0.010) were associated with discharge to healthcare services (Table 6).

The subgroup of those aged ≥65 with a reported fall injury had an OR of 3.08, indicating a 208% increase in referral to healthcare services (CI 95% 1.805, 5.260). The OR associated with Medicare as a primary payor source was 2.181 (CI 95% 1.204, 3.950), indicating a 118% increase in the probability of being referred to healthcare services. Those aged ≥65 in mTBI-S visits who reported Medicaid (*p* = 0.633) or Private (*p* = 0.451) as primary payor sources were not associated with referrals to healthcare services. The model fit for the stratified regression analysis was calculated as significant (*p* < 0.001); however, the Nagelkerke R Square was 0.039, indicating 3.9% variability in the outcome data that could be explained (Table 7).

## 4. Discussion

Previous research investigations have reported inadequate acute mTBI medical management [8] and diminished recovery [11,24,25,26,27] in older adults after mTBI. De Koning et al. and Marrone et al. have previously indicated that research was needed to identify determinants contributing to an increase in the probability of populations aged ≥65 not recovering after mTBI [24,27]. Existing studies have provided limited insight into populations aged ≥65 with mTBI and the relationships between falls, payors, and healthcare service referrals upon discharge from the ED [17,19,28]. The primary objective of this study, therefore, was to explore variables of populations aged ≥65, fall mechanism of injury, payor source, and associations in discharge outcomes among subsequent mTBI ED admissions. The research question was whether older adults (≥65 vs. <65), self-reported sex (female vs. male), fall-related injury (yes vs. no), and payor sources (Medicare, Medicaid, Private, or other) were associated with an increased probability of referral to healthcare services (health services vs. home) for people with subsequent mTBI ED admissions. The researchers further stratified the data into a subgroup of people aged ≥65 to determine if the findings were consistent and specific to older adults.

The research on geriatric populations and falls associated with healthcare service use in those returning to the ED for subsequent mTBI visits was limited. Explaining this evidence was critical to guide more tailored interventions, optimize discharge planning, and improve continuity of care in older adults with mTBI [4,11,29]. The Chi-squared findings of the current study were significant (*p* < 0.001, X^2^_1_ = 1142.2) in those aged ≥65 associated with healthcare service referral. Notably, although significant, these results alone could not discriminate what was contributing to the high X^2^ value. The fall injury variable had a significant association with health services (*p* < 0.001, X^2^_1_ = 123.6) compared to all other injuries in the population of people with subsequent visits to the ED following mTBI. The findings should be interpreted with caution due to the low percentage of reported fall injuries in the overall mTBI-S findings (4.2%) and in the stratified subgroup of older adults (7.5%). Although not generalizable due to the low percentage reported, these findings are consistent with Varriano et al.’s in that older adults and fall injury were significantly associated among Canadian participants with persisting mTBI symptoms [11].

The available literature examining the relationship between payor sources and discharge outcomes in populations aged ≥65 with subsequent mTBI visits was limited [14,17,19]. Understanding the payor source impact on post-ED care decisions has been essential for identifying disparities in accessing appropriate healthcare services [12,13,30,31]. In the current study’s overall mTBI-S sample, 37.4% reported Medicare as the primary payor source, whereas this proportion increased to 87.3% in the stratified subgroup of older adults. Of all of the payor sources in the mTBI-S sample, Medicare was significantly associated with healthcare service referrals (*p* < 0.001, X^2^_3_ = 1059.9). This differs from Seabury et al.’s research, where no association between the insurance source and healthcare service utilization after discharge was reported in all ages of those with mTBI [14].

Additionally, the present study’s findings aligned with studies that supported older adults with mTBI having a higher percentage of healthcare service referrals [17,19]. The logistic regression model for the current study, specific to mTBI-S, demonstrated that all payor variables did increase the probability of being referred to healthcare services with Medicare (OR 4.492, *p* < 0.001, CI 95% 3.423, 5.895), Medicaid (OR 1.984, *p* < 0.001, CI 95% 1.984, 2.551), and Private payors (OR 1.637, *p* < 0.001, CI 95% 1.273, 2.106). However, Medicare visits resulted in 4.49 times or a 349% increase in the probability of referral to healthcare services, whereas comparatively, Medicaid (98.4%) and Private payors (63.7%) demonstrated less of an increased probability of being referred to healthcare services. Additionally, the logistic regression model included an increase in probability for referral to healthcare services in the variables of age ≥ 65 (OR 4.172, *p* < 0.001, CI 95% 3.427, 5.079) and falls (OR 3.847, *p* < 0.001, CI 95% 2.649, 5.587). These results suggest that visits coded as age ≥ 65 had 4.17 times or 317% increased referral to healthcare services, and visits coded as fall injury had 3.85 times or 285% increased referral to healthcare services upon ED discharge. The stratified subgroup analysis corroborated these results in that older adults (aged ≥65) with fall-related injuries (OR 3.082, *p* < 0.001, CI 95% 1.805, 5.260) and Medicare as their primary carrier (OR 2.181, *p* = 0.010, CI 95% 1.204, 3.950) had a high probability of being referred to healthcare services. Although these results were not generalizable, they do highlight that older adults returning for subsequent mTBI were not associated with healthcare services if their reported payors were Medicaid (OR 1.293, *p* = 0.633, CI 95% 0.450, 3.713) or Private (OR 1.292, *p* = 0.451, CI 95% 0.664, 2.515). The results should be interpreted with caution, as the percentages were low for Medicaid (1.2%) and Private (8.8%) in the stratified older adult subgroup. The results raised the possibility that discharge planning for older adult populations may be influenced by payor source; however, further large sample studies would be needed to support the findings.

### Limitations

The present study’s findings must be interpreted within the context of its limitations. For example, ICD-10 codes recategorized into CCSR codes were used to identify the subsequent mTBI visits. To estimate the accuracy of the correct ICD-10 diagnosis codes, the investigators relied on previous study findings by Warwick et al. According to Warwick et al., an mTBI diagnosis, when listed as a primary diagnosis by the ED, had an estimated positive prediction of 96.9% (95% CI; 93.3%, 100%) [32]. However, it is worth noting that no validity studies have been conducted specifically for coding subsequent mTBI visits.

Although the model fit results in the current study were significant (*p* < 0.001, X^2^_6_ = 1434.5; Nagelkerke R Square 0.34), the study variables alone only explained 34% of the variance in the mTBI-S sample, and there was less explanation of variance (3.9%) in the stratified subgroup model among older adults (*p* < 0.001, X^2^_5_ = 48.8; Nagelkerke R Square 0.039). Additional characteristics outside of the current study variables were necessary to investigate a more significant model fit in prediction. Researchers have reported older adult multifactorial complexities and timeliness contributing to variance in ED discharge planning [13,30,33]. Previous research has included numerous factors to support prediction models for older adults regardless of mTBI diagnosis. For example, research on prediction models for ED discharge location among older adults has included predictors of sex, age, vital signs, arrival time, day of the week, ED wait times, payor source, past medical history (cardiovascular, pulmonary, cancer, obesity, diabetes, and number of chronic conditions), presenting injury (fracture, dislocation, back pain, or gastrointestinal bleeding) [33], prehospital location, number of medications, laboratory values [30], living situation, and autonomy in independence of activities of daily living [31]. Previous studies on mTBI and geriatric populations have accounted for personal and social factors related to persisting symptoms after mTBI [16,20,34,35,36]. The current study relied on the NEDS codes as the collected data of visits, which imposed constraints on incorporating additional variables, including caregiver and family structure [34,35], isolated versus non-isolated (co-morbid musculoskeletal injury) mTBI [16], and the patient’s prior level of function (psychological and cognitive status) associated with the visits [20,36]. These variables may impact the decision making in ED discharge planning. The NEDS dataset was limited in that the data were de-identified as visit counts and the diagnosis codes were recoded from ICD-10 codes into CCSR codes. Therefore, the investigators were unable to achieve the level of detail in the variables previously reported in retrospective research designs [13,30,31,33]. Of importance is that the present study gave insight into age, fall-related injury, payor source, and discharge referral relationships and provided potential implications for future research studies and healthcare models specific to older adults with mTBI returning to the ED.

The NEDS dataset contained visit information from ED admissions, and therefore, the investigators were unable to generalize the findings across patient care settings. The visit counts in the NEDS dataset had not furnished information regarding patient revisits to the ED or the progression of their admissions over time. Therefore, specific details regarding the timing of patient returns and the number of visits per patient were precluded. In fact, information about the number of returning visits and at what location the patient was initially diagnosed with mTBI was not available in the NEDS 2018 dataset [21]. Similarly, the NEDS dataset did not include medical settings outside of the ED, which may impact generalizability. Of importance is that the current study sample represented a comprehensive overview of the United States population, as the NEDS dataset accounted for 82.8% of the overall U.S. resident population and 82.2% of the total U.S. ED visits [21].

The investigators acknowledge that there were considerations that opened the potential for biases. For example, the investigators dichotomized age (<65, ≥65), converting a continuous variable into a categorical one, thereby reducing the statistical power and making the analysis less sensitive [23]. The investigators predefined these age groups to highlight the older adult population in subsequent mTBI ED admissions. An earlier study influenced the decision to group age (<65, ≥65) by reporting that participants with subsequent mTBI visits were significantly older (age mean, 50.4) compared to ages in the mTBI initial visit (age mean, 41.4) (CI 95%, *p* = 0.025) [22]. Another consideration of potential bias within the sample was the low report of falls in the overall mTBI-S sample (4.2%) and the stratified subgroup of older adults (7.5%). The fall-related injury findings may not be generalizable based on this study alone.

This study found that among all patients with mTBI-S, older adults were 4.17 times more likely to be referred to healthcare services. Fall-related injuries, although representing a low percentage of people, increased referral probability by 3.85, and Medicare coverage was associated with a 4.49 times higher referral rate. In the stratified analysis of older adults, fall-related injuries and Medicare coverage were still associated with higher referral rates, 3.08 and 2.18 times, respectively. Although low percentages were reported in the stratified sample, Medicaid and Private payor sources were referred at lower rates than those with Medicare, a disparity that warrants further investigation. The findings highlight potential inconsistencies in equitable healthcare service referrals based on age and insurance coverage in those with mTBI returning to the ED. By optimizing referral practices, the healthcare system can improve care continuity, reduce unnecessary utilization of acute services, and better allocate resources to support high-risk populations.

This study provided innovative and impactful contributions to both research and clinical practice related to mTBI in older adults, particularly those returning to the emergency department for subsequent visits. Unlike most studies that focused on mTBI incidents, this study uniquely investigated subsequent ED visits for mTBI, capturing a more vulnerable and complex patient population: those returning for additional care. This shift in focus provided insights into further investigation of recovery trajectories, persistent symptoms, and healthcare needs after initial mTBI. This study contributed novel data by stratifying to older adults, a group often underrepresented or treated as homogenous in mTBI research. This approach highlighted age-specific disparities and patterns in healthcare service referrals and supported previous research that emphasized the need to better understand outcomes in older adult populations [9,27]. Although fall injuries were underreported, the findings indicated a strong association between falls and increased referrals to healthcare services. This association underscored the need for further investigation into ED visits involving fall-related injuries, particularly concerning accompanying neurologic symptoms in older adults. Lastly, the inclusion of payor source (Medicare, Medicaid, Private, and other) as a variable and its association with discharge referral decisions was highly innovative. While previous studies reported no such associations [14], the findings of this study suggest that disparities in referrals based on insurance type warrant further investigation.

## 5. Conclusions

Previous research supports the idea that older adults (aged ≥65) experience prolonged recovery, decreased independence in daily activities, and diminished quality of life following mTBI. While mTBI in this population has been associated with increased costs, there has been limited research investigating factors associated with return visits to the ED. The findings of this study contribute to a broader understanding of ED return visits and discharge outcomes in older adults following mTBI, highlighting areas for future research. The results underscore the need to explore retrospective analyses examining fall-related-injury-reporting, associated consequences post-mTBI, payor reimbursement policies, discharge outcome effectiveness, co-morbidities, baseline functional status, vital signs, laboratory results at discharge, caregiver availability, and social support, which may inform value-based care models among older adult populations with mTBI. Prospective studies may evaluate ED interventions aimed at reducing persisting post-mTBI symptoms, including targeted patient education and a workflow for early clinical screening for older adults with acute mTBI. Ultimately, this may enhance post-discharge support and reduce the burden of mTBI in both patients and the healthcare system.

## Figures and Tables

**Table 1:** NEDS 2018 characteristics (N = 4932).

Variable		Visit Count	Percentage
Age	≥65	1793	36.4%
	<65	3139	63.6%
Sex	Female	2136	43.3%
	Male	2796	56.7%
Fall Injury	Yes	206	4.2%
	Other than fall	4726	95.8%
Payor	Medicare	1847	37.4%
	Medicaid	1157	23.5%
	Private	1230	25.0%
	Other (self-pay, no charge, or other)	690	14.0%
	Missing—payor	5	0.1%
Discharge Outcome	Home/no services	2914	59.1%
	Healthcare services	2015	40.9%
	Missing—discharge outcomes	3	0.1%

**Table 2:** Chi-squared analysis of subsequent mTBI visits and discharge outcomes and age, sex, falls, and payor source (N = 4932).

Variable		df	X^2^	*p*-Value
Age	≥65	1	1142.2	<0.001 ^1^
	<65			
Sex	Female	1	46.8	<0.001 ^1^
	Male			
Fall Injury	Yes	1	123.6	<0.001 ^1^
	No—other than fall			
Payor	Medicare	3	1059.9	<0.001 ^1^
	Medicaid			
	Private			
	Other (self-pay, no charge, or other)			

Abbreviations: df, degrees of freedom. ^1^ Significance *p* < 0.05.

**Table 3:** Logistic regression of discharge outcomes for subjects aged ≥65, female, with fall injuries and different payor sources (N = 4932).

Variable	Slope (B)	Standard Error	Wald	df	*p*-Value	Odds Ratio	CI (95%) Lower	CI (95%) Upper
Age (≥65)	1.428	0.1	202.5	1	<0.001 ^1^	4.172	3.427	5.079
Sex (Female)	−0.634	0.71	80.3	1	<0.001 ^1^	0.53	0.462	0.609
Fall injury	1.347	0.91	50	1	<0.001 ^1^	3.847	2.649	5.587
Medicare	1.502	0.139	117.4	1	<0.001 ^1^	4.492	3.423	5.895
Medicaid	0.685	0.128	28.5	1	<0.001 ^1^	1.984	1.543	2.551
Private	0.493	0.128	14.7	1	<0.001 ^1^	1.637	1.273	2.106

Abbreviations: df, degrees of freedom; CI, confidence interval. ^1^ Significance *p* < 0.05. Note: Payor (other) did not compute due to nonexistent combinations or ≤10 in the group.

**Table 4:** Model fit variables of age ≥ 65, sex, falls, and payor sources on discharge outcomes (N = 4932).

	Chi-Squared	df	*p*-Value	Nagelkerke R Square
Model	1434.5	6	<0.001 ^1^	0.34

^1^ Significance *p* < 0.05.

**Table 5:** Characteristics of stratified dataset of subjects aged ≥65 with mTBI (N = 1793).

Variable		Visit Count	Percentage
Sex	Female	793	44.2%
	Male	1000	55.8%
Fall Injury	Yes	135	7.5%
	Other than fall	1658	92.5%
Payor	Medicare	1564	87.3%
	Medicaid	22	1.2%
	Private	157	8.8%
	Other (self-pay, no charge, or other)	48	2.7%
	Missing—payor	2	0.1%
Discharge Outcome	Home/no services	505	28.2%
	Healthcare services	1288	71.8%

**Table 6:** Stratified logistic regression related to discharge outcomes in female subjects with fall injuries and different payor sources in populations aged ≥65 (N = 1793).

Variable	Slope (B)	Standard Error	Wald	df	*p*-Value	Odds Ratio	CI (95%) Lower	CI (95%) Upper
Sex (Female)	−0.395	0.107	13.6	1	<0.001 ^1^	0.674	0.546	0.831
Fall injury	1.125	0.273	17.0	1	<0.001 ^1^	3.082	1.805	5.260
Medicare	0.780	0.303	6.615	1	0.010 ^1^	2.181	1.204	3.950
Medicaid	0.257	0.538	0.227	1	0.633	1.293	0.450	3.713
Private	0.256	0.340	0.568	1	0.451	1.292	0.664	2.515

Abbreviations: df, degrees of freedom; CI, confidence interval. ^1^ Significance *p* < 0.05. Note: “payor (other)” did not compute due to nonexistent combinations or ≤10 in the group.

**Table 7:** Model-fit stratified variables of sex, falls, payor on discharge outcomes in populations aged ≥65 (N = 1793).

	Chi-Squared	df	*p*-Value	Nagelkerke R Square
Model	48.8	5	<0.001 ^1^	0.039

^1^ Significance *p* < 0.05.

## Data Availability

Restrictions apply to the availability of these data. The data were obtained from NEDS and are not available by these authors as per the data user agreement guidelines. The datasets were publicly available via https://hcup-us.ahrq.gov/tech_assist/centdist.jsp, and accessed on 9 December 2024.

## Abbreviations

The following abbreviations are used in this manuscript:

mTBI	Mild traumatic brain injury
mTBI-S	Mild traumatic brain injury, subsequent visits
ED	Emergency department
NEDS	National Emergency Department Sample
ICD-10	International Statistical Classification of Diseases and Related Health Problems
CCSR	Clinical classification software refined

## Appendix A

The appendix includes the collinearity diagnostics for the predictive variables of age ≥ 65, female sex, fall-related-injury status, and payor for supporting isolated relationships in the outcome variable of discharge outcomes (N = 4932).

**Table A1:** Collinearity diagnostics for predictive variables of age ≥ 65, female sex, falls, and payors for discharge outcomes (N = 4932).

Pearson’s Correlation ^1^			Collinearity Statistics		
**Age (>65)**	**Sex (Female)**	Fall	Payor (Medicare)	Tolerance ^2^	VIF ^3^
1.00	0.14	0.127	−0.515	0.73	1.371
0.14	1.00	0.10	−0.037	1.00	1.00
0.127	0.10	1.00	−0.098	0.98	1.02
−0.515	−0.037	−0.098	1.00	0.73	1.36
Variance Proportions					
Age (≥65)	Sex (Female)		Fall	Payor (Medicare)	
0.03	0.05		0.01	0.02	
0.03	0.01		0.78	0.02	
0.42	0.00		0.20	0.07	
0.04	0.87		0.00	0.07	
0.48	0.07		0.00	0.82	

Abbreviations: VIF, variance inflation factor. ^1^ Values of 1 = collinearity. ^2^ Values close to 0 = collinearity. ^3^ Threshold for collinearity conventionally “10”.

## Appendix B

The appendix includes the collinearity diagnostics for the predictive variables of female sex, fall-related-injury status, and payor for supporting isolated relationships in the outcome variable of discharge outcomes in people ages ≥ 65 (N = 1793).

**Table A2:** Collinearity diagnostics for predictive variables of age ≥ 65, female sex, falls, and payors for discharge outcomes (N = 1793).

Pearson’s Correlation ^1^			Collinearity Statistics		
Sex (Female)	Fall	Payor (Medicare)		Tolerance ^2^	VIF ^3^
1.00	0.01	−0.09		1.00	1.00
0.01	1.00	−0.32		0.98	1.02
−0.09	−0.32	1.00		0.73	1.36
Variance Proportions					
Sex (Female)		Fall		Payor (Medicare)	
0.06		0.03		0.04	
0.02		0.96		0.01	
0.69		0.00		0.22	
0.23		0.02		0.73	

Abbreviations: VIF, variance inflation factor. ^1^ Values of 1 = collinearity. ^2^ Values close to 0 = collinearity. ^3^ Threshold for collinearity conventionally “10”.
